# WHO’s Global status report on neurology: mixed messages for people with headache disorders

**DOI:** 10.1186/s10194-026-02281-7

**Published:** 2026-02-05

**Authors:** Timothy J. Steiner, Latifa Adarmouch, Amalie Berring-Uldum, Raquel Gil-Gouveia, Rigmor Jensen, Risako Shirane, Andreas Kattem Husøy

**Affiliations:** 1 NorHead, Department of Neuromedicine and Movement Science, Norwegian University of Science and Technology, Edvard Griegs gate, Trondheim, Norway; 2 Department of Neurology, University of Copenhagen, Copenhagen, Denmark; 3 Department of Brain Sciences, Imperial College London, London, UK; 4 Biosciences and Health Research Laboratory, School of Medicine and Pharmacy, Cadi Ayyad University, Marrakesh, Morocco; 5 Clinical Research Department, Mohammed VI University Hospital, Marrakesh, Morocco; 6 Department of Pediatrics and Adolescent Medicine, Copenhagen University Hospital – Herlev and Gentofte, Herlev, Denmark; 7 Hospital da Luz Headache Center, Neurology Department, Hospital da Luz, Lisbon, Portugal; 8 Center for Interdisciplinary Research in Health, Universidade Católica Portuguesa, Lisbon, Portugal; 9 Danish Headache Centre, Department of Neurology, Rigshospitalet Glostrup, Faculty of Health and Medical Sciences, University of Copenhagen, Copenhagen, Denmark; 10 Department of Neurology, Yale School of Medicine, New Haven, CT USA; 11 Department of Neurology and Clinical Neurophysiology, St Olavs University Hospital, Trondheim, Norway

**Keywords:** World Health Organization, Neurological disorders, Headache disorders, Health care, Health policy, Universal health coverage, Global Campaign against Headache

Releasing their *Global status report on neurology* [1] on 14 October 2025, the World Health Organization (WHO) accompanied it with the banner headline: “11 million lives lost each year: urgent action needed on neurological care”. The report, WHO claimed, provided “a comprehensive assessment of countries’ responses to neurological conditions”, setting “a baseline for monitoring progress under the *Intersectoral global action plan on epilepsy and other neurological disorders* (IGAP) [2] to improve brain health and reduce inequalities.”

The report was hailed as the first-of-its-kind, but with the somewhat disappointed rider that only 102 of 194 WHO Member States (53%) contributed to it. Although these countries represented 71% of the world population, this underwhelming response was an indicator – according to WHO – “of the limited attention given to neurology”.

This may be so. Within neurology, we ask, where are headache disorders?

Neurological conditions were defined broadly in the report as conditions affecting the nervous system, including communicable and vascular, which rather tilts the picture. The top contributors to death and disability were stroke and neonatal encephalopathy, but migraine was third. In 2021, age-standardized disability-adjusted life year (DALY) rates for migraine, per 100,000 people, were 380.0 for ages 5–19 years and 750.8 for ages 20–59 years; total rates for all neurological conditions were 1,705.4 and 3,443.1 respectively. Only stroke in the 20–59 years group (1,126.1) exceeded migraine as a cause of lost health; no other cause, except pre-term birth in the 5–19 group (234.3), came anywhere near. Epilepsy, the focus of IGAP [2], ranked seventh among the top contributors to death and disability, responsible for 185.1 and 174.0 DALYs per 100,000 people in these age groups.

It is the emphasis on mortality – evident in WHO’s banner headline – that pushes headache disorders down the health-priority pecking order. Headache disorders do not cause early death, but this means that *all* of the health loss associated with them is experienced by the living (with migraine, according to the most recent iteration of the Global Burden of Disease study, accounting for 90% of it [3]). Among the living, headache disorders lead all other neurological disorders by a large margin.

**Table 1:** Responses to WHO’s enquiry from 102 of 194 Member States by Region and income level (low versus high)

Global	African Region	Region of the Americas	Eastern Mediterranean Region	European Region	South-East Asia Region	Western Pacific Region	All low-income countries	All high-income countries
**Countries reporting standalone policies for headache disorders (** ***n*** **)**								
0	0	0	0	0	0	0	0	0
**Countries reporting that headache disorders are covered by awareness-raising campaigns or advocacy programmes (** ***n*** **)**								
13 (12.7%)	2	1	3	3	2	2	0	5
**Countries reporting that headache disorders are included in UHC benefits packages (** ***n*** **)**								
18 (17.6%)	2	5	2	6	1	3	2	10
**Countries reporting that social protection mechanisms are available for headache disorders (** ***n*** **)**								
23 (22.6%)	3	5	2	10	1	2	1	13
**Countries reporting that headache disorders are included in existing guidelines or standards (** ***n*** **)**								
29 (28.4%)	9	4	2	11	1	2	4	7
**Countries reporting that core indicators for headache disorders are integrated into the health information system and routinely collected (** ***n*** **)**								
18 (17.6%)	6	2	5	3	1	1	4	5
**Countries reporting that data for headache disorders have been compiled and reported (** ***n*** **)**								
23 (22.6%)	5	5	3	5	1	4	3	7

UHC: Universal health coverage

Member States could report on six tracer conditions: epilepsy, headache disorders (including migraine), meningitis, neurodevelopmental conditions, Parkinson’s disease and stroke [1]. Among 63 countries (32.5% of WHO Member States) with verified policies for any of these conditions, standalone policies included stroke (*n* = 6), epilepsy (*n* = 5), neurodevelopmental conditions (*n* = 4) and Parkinson’s disease (*n* = 3). None were specifically for headache disorders (Table 1). WHO’s enquiry related to targets specified in IGAP. Accordingly, it embraced awareness and advocacy, the provision of universal health coverage (UHC) benefits packages and of social protection mechanisms, the existence of national guidelines and standards, the integration and collection of core indicators for these disorders into countries’ health information systems, and the national compilation and reporting of data. Table 1 summarises the findings.

WHO’s enquiry also embraced the provision of essential medicines. Acetylsalicylic acid, ibuprofen and paracetamol, all on WHO’s essential medicines list (EML) for migraine [4], were, of course, widely available. Propranolol, the only migraine preventative drug on the EML [4], was available in 75% of responding countries. But sumatriptan, a specific antimigraine medicine developed well over 30 years ago and also on the EML, was reportedly available in only 34% of responding countries. Since it was the first triptan to market, and the first to become available generically, it is safe to assume no other triptan was more available.

While the report found that neurological conditions affected more than 40% of the global population, a key message was the “severe lack of qualified health professionals”. In particular, neurologists were “up to 82 times fewer per 100,000 people” in low-income countries than in high-income, putting, for many, “timely diagnosis, treatment, and ongoing care … simply out of reach.”

## Comment

For people with headache, these are mixed messages. Not one responding country reported a standalone policy for headache disorders. That, on its own, did not necessarily mean poor provision of care, or neglect of headache disorders in health and social security policies. With regard to awareness and advocacy, UHC benefits packages, and social protection mechanisms for headache disorders, the disparity between low- and high-income countries is unsurprising. But if these and the other indicators reflect the extent to which IGAP targets were met for headache disorders, it should be noted that, globally, only 28.4% of countries reported the existence (and, presumably, usage) of specific guidelines. All other IGAP targets were met by even fewer countries.

On the other hand, it is noteworthy that the African Region appeared to be doing rather well compared with all but the European Region – although the latter, largely composed of high-income countries, did not shine!

Here it should be emphasised that these were selected data, gathered from 102 countries (53% of Member States) that chose to respond. It might be thought that countries with better-developed neurological services were among those less likely to be dependent on WHO policy and guidance, and, consequently, among those less inclined to respond. The report referred to “notable regional variations”: submission rates were 30% in Western Pacific Region and 36% in South-East Asia Region, but 51–71% in the others; 50% for low-income countries and 55% for high-income. It is very unclear, therefore, what biases, if any, these might have introduced.

Does the report signal any likelihood of improvement in health coverage – and health – for people with headache? There are a few encouraging indicators in Table 1. But the report, pointing to the general neglect of neurological disorders, has very little to say about headache, despite identifying migraine as third highest cause of DALYs among neurological disorders. It highlights the lack of neurologists in low-income countries. In these ways, it appears rather negative. Our (*Lifting The Burden*’s) message would be that “timely diagnosis, treatment, and ongoing care” are *not* out of reach. For headache disorders, the requirement is not for neurologists, and these disorders (mostly) do not compete for scarce specialist services. What most people with headache need is advice and guidance on lifestyle management and, very importantly, on correct usage of over-the-counter medications, along with access to all medications on the EML (now including naproxen and eletriptan [5]). Much of this can be provided in primary care [6], while community pharmacists are an underutilised resource potentially able also to contribute materially [7].

## Data Availability

Not applicable.

## Abbreviations

DALY: Disability-adjusted life year
EML: Essential medicines list
IGAP: Intersectoral global action plan on epilepsy and other neurological disorders
UHC: Universal health coverage
WHO: World Health Organization
